# Enhancement of Functionality and Therapeutic Efficacy of Cell-Based Therapy Using Mesenchymal Stem Cells for Cardiovascular Disease

**DOI:** 10.3390/ijms20040982

**Published:** 2019-02-24

**Authors:** Chul Won Yun, Sang Hun Lee

**Affiliations:** 1 Medical Science Research Institute, Soonchunhyang University Seoul Hospital, Seoul 04401, Korea; skydbs113@naver.com; 2 Department of Biochemistry, Soonchunhyang University College of Medicine, Cheonan 34538, Korea

**Keywords:** mesenchymal stem cells, cardiovascular disease, exosome, natural products

## Abstract

Cardiovascular disease usually triggers coronary heart disease, stroke, and ischemic diseases, thus promoting the development of functional failure. Mesenchymal stem cells (MSCs) are cells that can be isolated from various human tissues, with multipotent and immunomodulatory characteristics to help damaged tissue repair and avoidance of immune responses. Much research has proved the feasibility, safety, and efficiency of MSC-based therapy for cardiovascular disease. Despite the fact that the precise mechanism of MSCs remains unclear, their therapeutic capability to treat ischemic diseases has been tested in phase I/II clinical trials. MSCs have the potential to become an effective therapeutic strategy for the treatment of ischemic and non-ischemic cardiovascular disorders. The molecular mechanism underlying the efficacy of MSCs in promoting engraftment and accelerating the functional recovery of injury sites is still unclear. It is hypothesized that the mechanisms of paracrine effects for the cardiac repair, optimization of the niche for cell survival, and cardiac remodeling by inflammatory control are involved in the interaction between MSCs and the damaged myocardial environment. This review focuses on recent experimental and clinical findings related to cardiovascular disease. We focus on MSCs, highlighting their roles in cardiovascular disease repair, differentiation, and MSC niche, and discuss their therapeutic efficacy and the current status of MSC-based cardiovascular disease therapies.

## 1. Introduction

Cardiovascular disease (CVD), including coronary heart disease, stroke, and ischemic diseases, is a global public health problem and accounts for more deaths of people worldwide than any other disease [1]. Ischemic diseases are caused by decreased blood flow in tissues or organs due to abnormal vascular conditions [2]. Pharmaceutical or surgical therapy is commonly used to treat these diseases. The application of pharmaceuticals, including thrombolytic agents and anti-inflammatory agents, are typical strategies for the cure of symptoms of these diseases [3,4]; however, the administration of these agents can lead to unexpected adverse reactions [5]. In addition, surgical approaches have also been used, but surgery entails a variety of disadvantages, such as surgical complications and disease recurrence [6]. Although pharmaceutical or surgical therapy may treat the functionality of vascular conditions, these therapies cannot enhance the regeneration and functional recovery of the surrounding tissues influenced by CVDs. Therefore, there is a need to develop effective strategies for treating CVD.

Human mesenchymal stem cells (MSCs) are isolated from various tissues, including bone marrow, adipose tissue, and the umbilical cord [7,8]. These cells can differentiate into bone, cartilage, muscles, tendons, and adipose tissue [9]. Furthermore, they can secrete several cytokines/growth factors that can modulate the immune response and increase the potential of expansion and differentiation of host cells, which may assist in the repair of damaged tissue [10,11]. Therefore, they are a promising cell source for tissue repair and treatment of various pathological conditions, such as myocardial infraction, stroke, and peripheral ischemic diseases [12,13,14]. For effective SC-based treatment in CVDs, it is important to increase the efficiency of SC-based therapy.

In this review, we first summarize the various types of CVDs and then explore the application of MSC-based therapy in CVDs and cite the underlying mechanism of therapy, the potential therapeutic effects of MSCs, and the enhancement of MSCs functionality.

## 2. Overview of Cardiovascular Disease

**Figure 1:**
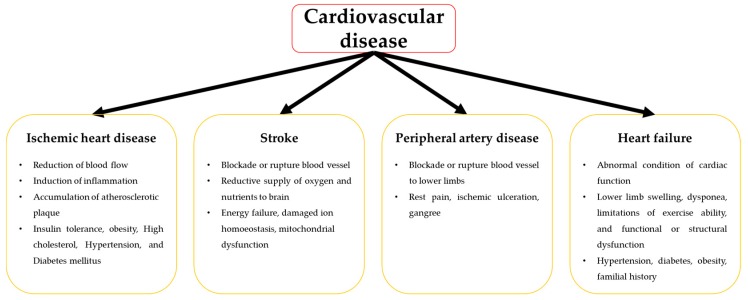
A schema illustrating the overview of cardiovascular disease with diverse symptoms.

CVD is a circulatory systemic disease caused by abnormal heart and blood vessel condition. CVD includes ischemic heart disease, stroke, peripheral arterial disease, and heart failure (Figure 1).

Ischemic heart disease (IHD) is caused by reduced blood flow to the heart and represents a major cause of morbidity and mortality [15]. Although the treatment of IHD has significantly improved, many patients still suffer from left ventricular dysfunction and heart failure. Patients with IHD can also exhibit insulin intolerance, high cholesterol, hypertension, diabetes mellitus, and obesity [16]. These symptoms are known as the pathology of metabolic syndrome, which induces inflammation and changes in platelet function leading to irregularity of vessel condition. Vessel inflammation affects cellular wall integrity and increases the accumulation of atherosclerotic plaques, which reduce the amount of blood flow to the heart [17,18].

Stroke is caused by the blockade, via a blood clot or by rupture, of a blood vessel that supplies oxygen and nutrients to the brain. When a stroke occurs, the severity of the brain ischemic event weakens the ability of the neuron to recover, and aggravates the disease on chronic strokes [19,20]. Acute ischemic stroke (AIS) is the most common type of stroke, comprising 85% of all strokes [19]. The pathophysiology of AIS is both simple and complex. Energy failure, damage of ion homeostasis, mitochondrial dysfunction, and induction of inflammatory responses occur during AIS. AIS also induces cell death via necrosis and apoptosis [21]. Ischemic stroke occurs in two major damage zones, the core ischemic area caused by instant cell death via necrosis, and the peri-infarct penumbra area caused by delayed programmed cell death [22]. Due to the delayed cell death, it may be possible to salvage the peri-infarct penumbra with the appropriate therapeutics.

Peripheral artery disease (PAD), which displays symptoms of narrowed or blockaded blood vessels preventing blood flow to the lower limbs, is caused by atherosclerosis and thrombosis. PAD is a major health problem associated with dysfunction and limb loss, and is a significant predictor of CVD and mortality [23]. Critical limb ischemia (CLI) is a severe symptom of PAD, and presents with rest pain, ischemic ulceration, or gangrene with or without tissue damage [24,25]. CLI is a complex process commonly caused by atherosclerosis, which involves macrovascular and microvascular dysfunction. The consequences of macrovascular dysfunction are obstructive lesions and thrombosis [26] that result in a significant decline in blood flow to the damaged limb and lead to CLI symptoms, such as rest pain and ischemic ulcers [27,28]. Microvascular dysfunction is a symptom of CLI that leads to changes in the structure and function of endothelial cells, which causes microcirculatory alterations and a weakened and less effective tissue oxygen exchange [29].

Heart failure is a symptom of abnormal cardiac function, with failure in blood supply to the peripheral tissues to provide an adequate amount of blood and oxygen to meet the required demand of metabolism [30,31]. Heart failure includes several symptoms, such as lower limb swelling, dyspnea, and limitations of exercise ability caused by structural or functional problems of the cardiac function [32]. Furthermore, heart failure is caused by diverse risk factors including hypertension, diabetes, obesity, and familial history of heart failure. These risk factors result in cardiac damage and progressively worsen cardiac functionality until the development of end-stage heart failure [33]. Finally, heart failure is a considerable cause of morbidity and mortality and deteriorates the quality of life and cardiac functionality.

Over the past two decades, many therapeutic advances in CVD have been accomplished in the field of medical and surgical treatments [3,34,35,36]. Despite these advances in CVD treatment, a new therapeutic strategy is still in demand for the treatment of CVD, and also for the accompanied conditions affected by ischemia. MSCs might be a potential cell source for the treatment of CVD, because they can be isolated easily from various tissues, such as bone marrow, adipose tissue, and the umbilical cord, and have the capacity for multipotent differentiation and self-renewal [7,8,37]. In addition, stem cells have paracrine effects; they can secrete bioactive factors regulating immunomodulation and angiogenesis, which may promote damaged tissue repair and regeneration [10,11,38,39]. Therefore, stem-cell-based therapy is considered the best promising treatment for the repair of damaged and surrounding tissues affected by CVD.

## 3. Diverse Stem Cell Use for the Treatment of Cardiovascular Disease

Embryonic stem cells (ESCs) are totipotent and have the capacity to differentiate into cells that derive from three germ layers: ectoderm, endoderm, and mesoderm [40]. It has previously been observed that ESCs have the ability to differentiate into functional cardiac, neuronal, and pancreatic cells [41]. ESCs can be differentiated into cardiomyocytes [42,43] that display a cell morphology and physiology similar to adult cardiac cells. Moreover, ESC-derived cardiomyocytes express diverse cardiac-specific genes and transcription factors, such as guanine‒adenine‒thymine‒adenine binding protein 4 (GATA4) and NK2 homeobox 5 (NKx2.5) [44,45]. In addition, ESC-derived cardiomyocytes can respond to pharmacologic agents via receptors specific to cardiomyocytes [45]. One study observed that models of transplanted ESCs in vivo improved the cardiac functionality in ischemic-damaged rat hearts [46]. The treatment of CVD using ESCs is a promising potential therapeutic strategy, but there is an issue of controversy with medical ethics due to their origin, the possibility of teratoma formation, and the immune rejection in humans, thus limiting their usage.

The induced pluripotent stem cells (iPSCs) are generated from adult cells, like fibroblasts, by reprogramming regulating transcription factors, such as Oct4, Sox2, Klf4, and c-Myc [47,48,49], and can be differentiated into a variety of cell types in humans. Some studies indicated that iPSCs are analogous to the diverse characteristics of ESCs, such as morphology, surface markers, gene expression, and teratoma formation in vivo, and have the same cell culture conditions as ESCs [48,50]. One study demonstrated that iPSC-derived cardiomyocytes could be used to significantly improve cardiac function in a mini pig myo-cardiac infarction model [51]. Another study has shown that combining iPSCs cell sheets with the omental flap technique led to an enhanced cell survival rate of the transplanted iPSCs [52]. Therefore, iPSCs are considered an alternative resource of cell-based therapy for CVD. The advantages of iPSCs include their easy generation, compared with the adult stem cells and ESCs, and low risk of immune rejection during CVD treatment [48,53]. Furthermore, iPSCs may escape the ethical and immunological rejection problems related to the usage of ESCs [54,55].

**Figure 2:**
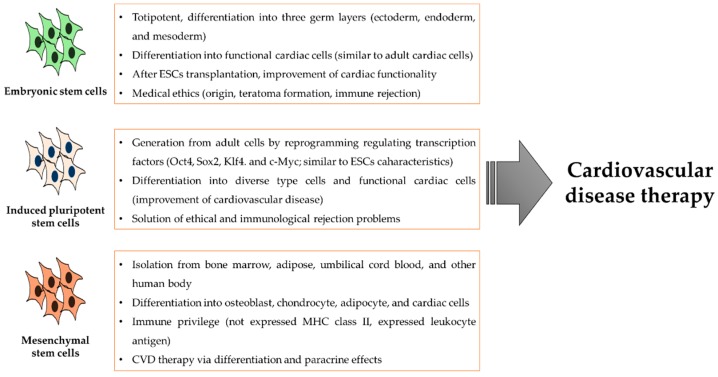
A diagram of diverse stem cells for the treatment of CVD.

Mesenchymal stem cells (MSCs) are a heterogeneous cell population that was first detected in the bone marrow, and then in other tissues, including adipose tissue and umbilical cord blood [56,57]. MSCs express a group of specific surface markers, such as CD44, CD73, and CD90 [58]. Under suitable conditions, MSCs have the ability to differentiate into osteoblasts, adipocytes, and chondrocytes [59]. In addition, MSCs can differentiate into cardiomyocytes in vitro and in vivo [60,61,62,63]. Many studies have indicated that MSCs can be immune privileged in both human and animal models due to the absence of expression in MHC class II molecules and the presence of expression in human leukocyte antigen and histocompatibility class I molecules [64,65,66,67]. An advantage of the MSC-based therapy to treat CVD is that these cells are easy to obtain from diverse tissues [68,69]. Furthermore, many studies have demonstrated that MSCs can survive and differentiate in allograft and xenograft animal models without the risk of immune rejection [70,71,72,73] (Figure 2).

## 4. The Potential Therapeutic Effects of Mesenchymal Stem Cells in Cardiovascular Disease

MSCs are used for stem cell therapy for CVD because they have the capacity to differentiate into diverse types of cells in vitro and in vivo [74,75,76]. Unlike other stem-cell-based therapies, MSCs do not require differentiation into a mature cell type prior to administration and have strong homing capacities in the damaged sites after cell transplantation [9,77]. Some studies have demonstrated that intravenously injected MSCs can migrate specifically to the sites of inflammation caused by ischemic damage [78,79]. Shin et al. have demonstrated that administration of MSCs following myocardial infarction (MI) injury enhanced the production of adenosine via CD73 activity, and attenuated ROS mediated inflammatory responses [78]. In addition, Matthew et al. have indicated that intravenous injection of MSCs after acute MI improved the myocardial function due to mobilization and homing of MSCs in the damaged sites [79]. Another advantage of MSC-based therapy is that allogeneic cells do not display severe issues of immune rejection [80,81]. A recent study has suggested that the immunomodulation of MSCs is related to IDO expression, a weaken immune response through suppression of T cell proliferation and function. Furthermore, MSCs attenuate host immune activity by secreting anti-inflammatory factors, and decreased inflammatory responses at the damaged sites [81]. In a clinical trial, Hare et al. indicated that allogeneic and autologous MSCs are safe and effective for treating ischemic heart disease, and allogeneic MSCs did not stimulate a donor-specific immune reaction [82].

Many preclinical investigations have utilized MSCs, and demonstrated their significant advantageous effect on damaged tissue structure and function. For example, one study demonstrated that MSCs could alleviate cardiac injury and promote recovery of heart function via neovascularization by releasing angiogenic factors, but not direct contribute in animal models of ischemic heart disease [83]. Another study confirmed that administration of allogeneic MSCs to the surrounding site of myocardial infarction in an animal model resulted in significant reduction in the infarct zone and significant improvement in left ventricular volumes [84,85,86,87]. Furthermore, other studies demonstrated that the administration of allogeneic MSCs to an infarcted myocardium in an animal model resulted in enhancement in local contractility and myocardial blood flow, as well as engraftment, differentiation, and enhanced survival rate in a large animal model [88,89,90].

Several studies demonstrated that MSCs therapy is significantly improving the symptoms of CVD via angiogenesis, repair of damaged areas, and enhancement of myocardial function; the application of MSCs therapy is a promising therapeutic strategy to CVD treatment. However, some evidence exists that the therapeutic effects of MSCs do not directly contribute at injurious sites. Therefore, these results lead to the paracrine hypothesis that MSC-based therapy produces factors that act regionally or systemically to favorably impact recovery.

## 5. The Treatment of Cardiovascular Disease via Mesenchymal Stem-Cell-Derived Exosomes

Exosomes are secretory vesicles of 30–100 nm diameter derived from plasma membrane and multivesicular endosomes, and released into the extracellular environment. Some studies confirmed that exosomes could contain cytokines, proteins, mRNAs, miRNAs, and rRNAs [91,92,93]. Exosomes may be released by a variety of cells including T cells [94], B cells [95], mast cells [96], platelets [97], and tumor cells [98,99]. For example, mast-cell-derived exosomes enhanced the proliferation and differentiation of T cells [96]. The exosomes derived from cancer cells can transfer molecular and genetic information from tumor cells to normal or other abnormal cells residing at the surrounding or at distant regions [98]. Therefore, exosomes are important for the intercellular communication by transferring proteins, mRNAs, and miRNAs to the nearby cells inducing coordinative function in the organisms [100,101,102].

MSCs synthesize and secrete functional exosomes that consist of phospholipid vesicles. In MSC-derived exosomes, many proteins have been detected by liquid chromatography‒mass spectrometry/mass spectrometry [103]. These proteins, depending on their functions, indicated that exosomes have the potential to influence many biological processes, such as angiogenesis, and the inflammation related pathway [104]. This effect is consistent with the reported efficacy of MSCs for the treatment of many diseases. MSC-derived exosomes were first examined in 2010 in a myocardial ischemia/reperfusion injury mouse model [105] and it was confirmed that the administration of MSCs-derived exosomes to a stroke rat model alleviated the symptoms via promotion of angiogenesis, neurite remodeling, and neurogenesis [106]. Moreover, another study indicated that MSC-derived exosomes reduced ROS production and enhanced autophagy via AMPK/mTOR and Akt/mTOR pathways, reduced apoptosis and myocardial infarct size, and improved heart function in animal models of myocardial ischemia reperfusion injury [107].

miRNAs are endogenous and conserved small non-coding RNAs that interact with mRNAs and associate with enhancement of mRNA degradation, translational suppression, and regulation of gene expression [108]. miRNAs are considered as crucial regulators of cellular processes, including proliferation, differentiation, apoptosis, and metabolism. A recent study reported that MSCs have protective effects in CVD via exosomal miR-22 targeting methyl CpG binding protein 2 (*Mecp2*) to decrease apoptosis [109]. Another study suggested that miR-221 in MSC-derived exosomes contributed to an anti-apoptotic effect by suppressing p53-upregulated modulator of apoptosis (PUMA), which enhanced the cardioprotective effects [110]. Moreover, the exosomal miR-21 derived from MSCs regulates the inhibition of anti-apoptotic proteins and increases the angiogenic proteins via the activation of phosphatase and tensin homolog (PTEN), and the Akt pathway, and then improves the therapeutic effects of MSC-derived exosomes in the MI model [111].

Some studies have demonstrated that MSC-derived exosomes are associated with the inhibition of inflammation responses and can therapeutically benefit CVDs [112,113]. It is reported that exosomes derived from MSCs significantly promoted tube formation of human umbilical vein endothelial cells (HUVECs) and attenuated the function of T cells via suppression of cell proliferation in vitro. The administrated exosomes in a rat myocardial infarction model reduced infarct size and protected cardiac function via improvement of the neovascularization and blood flow [114]. In addition, another study showed that MSC-derived exosomes relieved inflammatory response, reduced infarct area, and promoted cardiac function in hearts after ischemia reperfusion injury [113].

**Figure 3:**
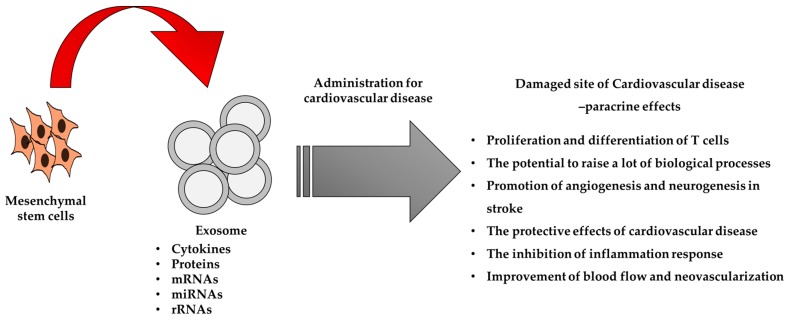
A schematic illustration of the roles of MSC-derived exosomes in the treatment of CVD via paracrine effects.

In addition, angiogenesis is one of the diverse processes affected by exosomes, a process related to the proliferation, differentiation, and migration of endothelial cells [115,116]. Adipose MSCs (AdMSCs) can generate extracellular vesicles including exosomes and micro-vesicles, which contain abundant pro-angiogenic molecules. The effects of exosomes derived from AdMSCs are significantly associated with potential effects of angiogenesis both in vitro and in vivo [117]. AdMSC-derived exosomes significantly increased tube formation of endothelial cells, and the subcutaneous injection of HUVECs pretreated with exosomes enhanced angiogenesis in a nude mouse model. Additionally, administration of AdMSC-derived exosomes promoted angiogenesis in the peri-infarct site and maintained cardiac function in an ischemic heart disease mice model [118]. Moreover, HUVECS can uptake MSC-derived exosomes, resulting in an increase in the functionality of endothelial cells. Therefore, using MSC-derived exosomes there is promotion of blood flow recovery, and then alleviation of infarct size and protection of cardiac performance in an acute myocardial infarction rat model [119]. A consistent observation suggested that the exosomes derived from MSCs have the significant advantage of stimulating anti-apoptosis, anti-inflammation, and neovascularization. These results lead to the paracrine effects of MSC-derived exosomes associated with CVD recovery (Figure 3).

## 6. The Treatment of Cardiovascular Disease via Mesenchymal Stem Cells Enhanced Natural Products

Stem cell therapy has rapidly progressed in the treatment of cardiac function in animal models of myocardial infarction and in humans with ischemic cardiovascular disease [120]. Recent studies have demonstrated that the transplantation of bone marrow MSCs enhanced the functional recovery and reduction of damaged sites in the ischemic cardiovascular disease [121,122,123]. Many animal and clinical studies have evidenced that cell therapy with MSCs is able to restore the cardiac function after myocardial infarction, potentially via angiogenesis and myogenesis [124,125]. However, the therapeutic effect of MSC transplantation is limited by its low viability in the damaged area post-transplantation. For example, many of the MSCs administrated into the left ventricle of the adult murine heart died within one week post-injection [126]. This indicated that the ischemic microenvironment of the infarcted myocardium provided poor conditions for MSC survival. Many studies have also shown that the differentiation of implanted MSCs into cardiomyocytes can enhance cardiac function during acute myocardial infarction [127,128]. Therefore, increasing the survival and differentiation of implanted MSCs after transplantation is an important factor in successful cellular therapy.

A natural product, *Gingko biloba* (EGb) leaf, has been used as a traditional Chinese medicine for a long time. EGb 761, an extract from *G. biloba* leaf, has been developed and consumed as a dietary supplement and an herbal remedy [129]. A previous study indicated that treatment with EGb 761 significantly reduced the number of infiltrated inflammatory cells in a myocardial infarction mouse model. The EGb 761 treatment increased the activity of antioxidant enzymes, SOD and catalase. The administration of EGb 761 also had a protective effect on myocardial infarction-induced MSC apoptosis during MSC transplantation. Furthermore, EGb 761 treatment increased the differentiation of MSCs into cardiac cells after MSCs transplantation [130].

Another natural product, Suxiao jiuxin pill (SJP), consists of two major components, tetramethylpyrazine (TMP) and borneol (BOR), and is a prominent traditional Chinese medicine used to treat acute ischemic heart disease [131,132,133]. SJP has significant effects on oxidative stress and vascular reactivity that may lead to improved blood flow. The action of SJP is to increase exosome release via Rab27, a small GTPase in the Rab family, and control the exosome secretion in mouse cardiac MSCs [134]. In addition, the SJP-treated MSC-derived exosome downregulated the expression of the demethylase UTX, then regulated the expression levels of H3K27me3 associated with histone remodeling, and finally promoted the proliferation of the mouse cardiomyocytes. These findings indicated the potential therapeutic effects of SJP to treat CVD through the enhancement of MSCs functionality [135].

Tauroursodeoxycholic acid (TUDCA) is an endogenous hydrophilic tertiary bile acid that exists in humans at low levels. Recent studies have confirmed that TUDCA alleviated the symptoms of a variety of diseases, including neurodegenerative diseases, vascular diseases, and diabetes [136,137,138]. TUDCA treatment of AdMSCs reduced the activation of ER stress, which in turn would induce apoptosis. In addition, the treatment of TUDCA increased the expression of PrP^C^, regulated by Akt phosphorylation, and increased antioxidant effects in AdMSCs. The transplantation of TUDCA-treated AdMSCs enhanced the blood perfusion ratio, vessel formation, and transplanted cell survival in a murine hindlimb ischemia model [139].

Melatonin is an endogenously secreted indoleamine hormone generated by the pineal gland [140]. Melatonin is secreted by a variety of tissues, including the bone marrow, liver, and gut [141]. Melatonin can enhance proliferation, resistance to oxidative stress, and confer immunomodulatory properties in AdMSCs; the upregulation of PrP^C^ promotes MSC proliferation and self-renewal. In addition, melatonin regulates the immunomodulatory effects of AdMSCs. In a murine hind-limb ischemia model, AdMSCs pretreated with melatonin improved blood flow perfusion, limb salvage, and vessel regeneration via reduction of infiltrating macrophages and apoptosis in the affected local cells and transplanted AdMSCs. These results indicated that melatonin promotes MSCs functionality and neovascularization in ischemic tissues [39].

## 7. Conclusions

Experimental evidence and clinical trials have demonstrated the feasibility, safety, and efficiency for CVD therapy from diverse origins and tissue-derived MSCs (Table 1), but there is still uncertainty about the real efficacy of MSCs on promoting engraftment and accelerating the recovery of CVD. Table 1 shows that several types of MSCs are used as therapeutic tools of cardiovascular disease and ischemic disease due to the multiple functionalities of MSCs. However, despite the high therapeutic potential of MSCs, their application is limited because of the low survival rate in harsh conditions of damaged areas by CVD, such as inflammation, oxidative stress, and restriction of nutrients [142,143]. Furthermore, when MSCs are isolated from patients with CVD for use as autologous MSCs, their function is generally decreased due to deterioration of the patient’s health [144]. Therefore, it is necessary to develop a novel method for enhancing the therapeutic efficacy of MSCs under pathophysiological condition.

In this review, we suggest various strategies to improve MSCs’ functionality in order to increase their therapeutic effects for CVD. Since the characteristics of MSC are significantly different depending on age, sex, origin of MSC, status of MSC senescence, health condition, and stage of CVD [145,146,147,148], it is also important to apply a proper methodology for enhancing the therapeutic potential of patient-specific MSCs.

## Figures and Tables

**Table 1:** A summary of the effects of MSCs in the treatment of CVD.

Pathological Condition	Type of Source	Findings	Reference
Acute MI	BM-derived MSC	Increase of adenosine via CD73 activity, reduction of inflammatory responses, mobilization, homing of MSCs, reduction of infarct sites, improvement of cardiac function	[78,79,84,85,86,88,89]
Ischemic disease	Ad-derived MSC	Immunomodulation, reduction of T cell proliferation and function, anti-inflammatory effects	[81]
Ischemic disease	BM-derived MSC	Neovascularization, recovery of damaged cardiac muscle	[83]
MI/R	ES-MSC derived exosome	Recovery of tissue injury, protection of cardiac function, reduction of immune response	[105,113]
Stroke	BM-MSC derived exosome	Promotion of angiogenesis, neurite remodeling and neurogenesis	[106]
MI/R	BM-MSC derived exosome	Reduction of ROS production, apoptosis, infarct size, enhancement of autophagy, promotion of HUVEC function and angiogenesis, immunomodulation	[107,114,119]
MI	miR-22/Mecp2	Reduction of apoptosis and cardiac fibrosis	[109]
Cell ischemic injury	miR-221	Anti-apoptotic activity, cardioprotective effect	[110]
MI	miR-21	Anti-apoptotic activity, neovascularization	[111]
MI	*Gingko biloba*	Reduction of immune responses, antioxidant activity, anti-apoptotic activity, differentiation of MSCs	[130]
In vitro	Suxiao jiuxin pill	Increase of exosome releases, histone remodeling, promotion of cardiomyocyte proliferation	[134,135]
Ischemic disease	TUDCA	Reduction of ER stress, antioxidant activity, improvement of ischemic injury site	[139]
Ischemic disease	Melatonin	Enhancement of proliferation, antioxidant activity, immunomodulation	[39]
